# Exploring the agreement between diagnostic criteria for IBS in primary care in Greece

**DOI:** 10.1186/1756-0500-1-127

**Published:** 2008-12-03

**Authors:** Foteini Anastasiou, Ioannis A Mouzas, Joanna Moschandreas, Elias Kouroumalis, Christos Lionis

**Affiliations:** 1 Clinic of Social and Family Medicine, Department of Social Medicine, Faculty of Medicine, University of Crete, Heraklion, Crete, Greece; 2 Department of Gastroenterology, University Hospital of Heraklion, Heraklion, Crete, Greece; 3 Biostatistics Laboratory, Department of Social Medicine, Faculty of Medicine, University of Crete, Heraklion, Crete, Greece

## Abstract

**Background:**

Irritable Bowel Syndrome (IBS) is frequently diagnosed in primary care. Its diagnosis is based on diagnostic criteria but their use is limited in primary care.

We aimed to assess the diagnostic agreement between the older (Manning's and Rome II) and the new (Rome III) criteria for the diagnosis of IBS in primary care in Greece.

**Methods:**

Medical records of 5 Health Centers in rural Crete, Greece, were reviewed for a four-year period and patients with the diagnosis of IBS were invited to a structured interview. Kappa agreement of the Rome III criteria with the criteria of Manning and Rome II was estimated. One hundred and twenty three patients were eligible for interview and 67 (54.5%) participated. Forty-six (69%) fulfilled the Manning, 32(48%) the Rome II, and 16(24%) the Rome III criteria. Twenty-seven (40%) patients were identified as IBS according to the questionnaire for the identification of functional gastrointestinal diseases (FGIDs). The agreement of Rome III with Manning criteria was poor (kappa = 0.25). The agreement between the FGIDs questionnaire and the Manning, Rome II and Rome III criteria was: kappa = 0.30, 0.31 and 0.24 respectively. Moderate agreement was found between the Rome II and III criteria (kappa = 0.51).

**Conclusion:**

Questionnaires and criteria deriving from expert's consensus meetings or tertiary hospitals are not easy to apply in rural primary care where symptoms are often underestimated by patients and complicated questions can be confusing.

## Background

Irritable Bowel Syndrome (IBS) is frequently diagnosed in primary care. [1] During the last decades efforts to provide reliable diagnostic criteria for IBS have been undertaken, starting with the criteria of Manning [2] and the consensuses of Rome I, II and III. [3,4] Classification criteria such as Rome II developed through experts consensus may be less applicable to primary care IBS patients [5] and their implementation in primary and secondary health care settings does not seem to be widely adopted. [6,7]

In Greece the subject of functional gastrointestinal disorders in primary health care seems to be neglected.[8,9] A recent study in rural Crete revealed that primary care physicians failed to diagnose these disorders.[8] This cross-sectional study led to the development of a database of patients with IBS. The advent of the new consensus (Rome III) on the diagnosis of IBS was an important incentive to explore to what extent the application of the new standards alters the diagnosis previously made within the primary care setting in Crete. This paper seeks to explore issues of diagnostic suitability and applicability of different classification criteria when they are used for IBS patients in primary care.

## Methods

### Setting and study population

The medical records of four Primary Health Care (PHC) centers and one primary surgery were reviewed from March 1996 till February 2000 with a methodology explained elsewhere.[8] All the patients with the diagnosis of IBS or spastic colitis or functional disorders of the large bowel were pooled together as IBS patients (ICPC 2: D93/ICD10: K58). The estimated occurrence rate of the IBS patients in this cross-sectional study was 1.2 per 1000 person-years. [8] This low IBS rate was attributed to the free access that Greek patients have to public health services without prior referral from their primary care centre. It was also uncertain to what extend patients with IBS were experiencing minor symptoms and thus they did not seek medical care from their primary care physicians. [8] Patients with IBS in this Cretan database were mostly women older than 70 and this fact can explain the high occurrence rate of IBS in people older than 65 years. However, both findings from this report need to be verified in future studies in this region.

All the identified IBS patients were considered eligible for a structured interview.

### Instruments

Each of the eligible patients was personally invited to a semi-structured interview. All interviews were performed by the same researcher during scheduled home visits and were based on a detailed personal and family history questionnaire. Co-morbidity and medication were documented both through direct questions during the interview and by patient's personal insurance book. The Manning criteria for IBS and the Rome II criteria for IBS and dyspepsia were applied. [2,3]

The questionnaire for the identification of dyspepsia in the general population (IDGP), which was translated and validated into Greek [10,11] was applied in order to document co-morbidity with dyspepsia and GERD. It consists of 11 main questions answered by yes or no, on upper gastrointestinal symptoms together with frequencies and consultation behavior, and one open question. The questionnaire for the identification of functional gastrointestinal disorders (FGIDs) [12] was also used. This questionnaire based on the Rome I criteria through nine different sets of questions provides a detailed picture of patients gastrointestinal problems. Main questions on symptoms duration from this questionnaire combined with Rome's II three main diagnostic criteria extended our comparison towards Rome III criteria retrospectively. All the diagnostic criteria and the questions used for the Rome III are shown in Table 1.

**Table 1:** All diagnostic criteria for IBS and the questions matching Rome III

**Manning Criteria**
Abdominal pain with 2 or more of the following:
1. Abdominal pain relieved by defecation; and/or
2. Abdominal pain onset associated with more frequent stools; and/or
3. Abdominal pain associated with looser stools; and/or
4. Abdominal distension or bloating; and/or
5. Feeling of incomplete defecation; and/or
6. Mucus in stools (Br Med J 1978)
**Rome II Criteria for IBS**
At least 12 weeks or more, which need not be consecutive, in the preceding 12 months, of abdominal discomfort or pain that has 2 out of 3 features:
1. Relieved by defecation
2. Onset associated with a change in frequency of stool
3. Onset associated with a change in form (appearance) of stool
**Symptoms that Cumulatively Support the Diagnosis of IBS:**
1. Abnormal stool frequency (may be defined as greater than 3 bowel movements per day and less than 3 bowel movements per week);
2. Abnormal stool form (lumpy/hard or loose/watery stool);
3. Abnormal stool passage (straining, urgency, or feeling of incomplete evacuation);
4. Passage of mucus;
5. Bloating or feeling of abdominal distension. (Gut. 1999)

**Rome III**
Recurrent abdominal pain or discomfort at least 3 days per month in the last 3 months associated with *2 or more *of the following:
1. Improvement with defecation
2. Onset associated with a change in frequency of stool
3. Onset associated with a change in form (appearance) of stool
*Criteria fulfilled for the last 3 months with symptom onset at least 6 months prior to diagnosis*.(Gastroenterology 2006)

**Rome III matching questions from the interview**
Abdominal discomfort or pain
1. Relieved by defecation
**2. **Onset associated with a change in frequency of stool
3. Onset associated with a change in form (appearance) of stool
**(Rome II)**
1. "How many times per week do you experience the symptoms? (1 per week/less frequent/more frequent)". *Patients who answered that they experienced the symptoms less than one time per week were considered as negative for the Rome III criteria*.
2. "For how long have you been experiencing the symptoms? (1 year/2 years/5 years)". *When patients answered that they had been experiencing the symptoms for less than a year the duration was noted (in months)*.
(**FGIDs questionnaire**)

### Statistical analysis

Comparisons of the characteristics of participants and non-participants were made using the chi-squared test for categorical variables and the non-parametric Mann-Whitney test for possible age differences, as age appeared negatively skewed in each group. In the FGIDs questionnaire age is a criterion for the differential diagnosis of organic disease against IBS thus no comparison with age was performed for this questionnaire. The chance-corrected agreement between the Manning and the Rome II criteria compared with the new Rome III criteria was estimated using Cohen's kappa [13]. Confidence intervals were calculated using the asymptotic variance, based on the normal approximation to the distribution of the kappa statistic [14]. Strength of agreement was interpreted using the following categories: < 0.20 poor, 0.20–0.40 fair, 0.41–0.60 moderate, 0.61–0.80 good, over 0.80 very good [14] Possible age and sex differences between the proportions classified with IBS using the three criteria (Manning's, Rome II, and Rome III) were assessed using the Mann-Whitney test and Fisher's exact test respectively. Confidence intervals for single proportions, and for differences between proportions, were calculated using the normal approximation to the binomial distribution. SPSS version 15 was used for all statistical analyses (SPSS for Windows, release 15.0.0, and 6/9/2006. Chicago: SPSS Inc). The significance level was set to 5%.

### Ethics

This study was approved by the Ethical Committee of the University Hospital of Heraklion, Crete, Greece (RN: 7173/2000). All participating patients were informed about the purposes of the study and gave their consent.

### Participation

**Figure 1:**
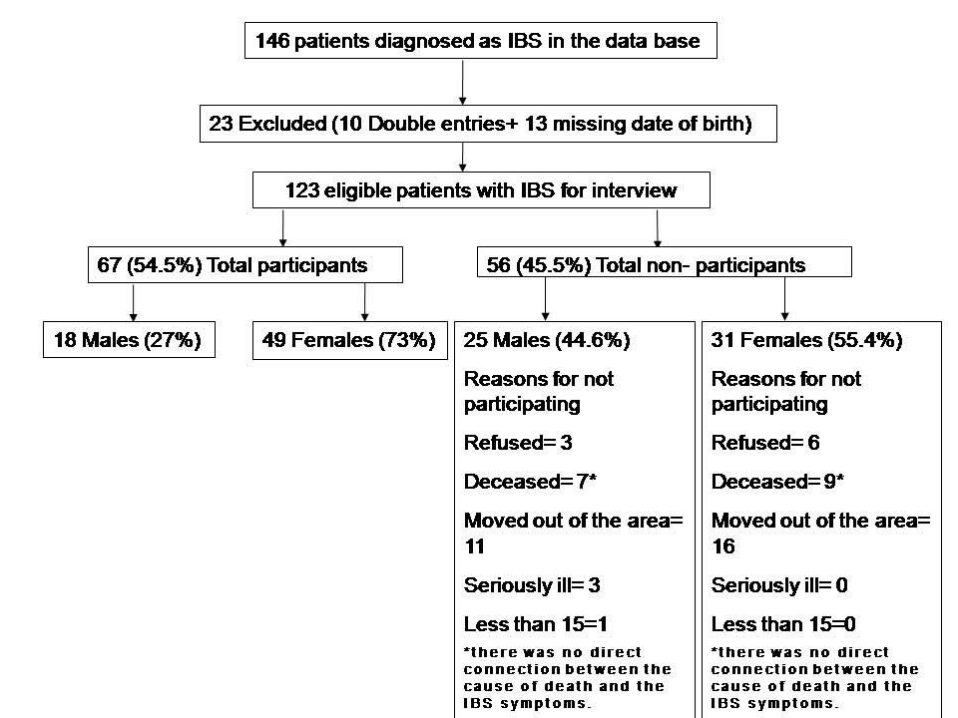
Flow chart of IBS patients.

The original database included 146 patients identified with the diagnosis of IBS. [8] Ten double entries were located. For thirteen entries, no date of birth was available. These patients were excluded due to the high possibility of synonymies. Finally, 123 patients were contacted for interview. Sixty-seven patients participated in the interview (54.5%). A flowchart including reasons for non-participation is shown in Figure 1. The mean interval period between the original doctor's diagnosis and the interview was 6.4 (SD: 1.24) years. The characteristics of patients with IBS according to participation status are presented in Table 2. Age distribution was not found to differ between the two groups (Mann-Whitney z = -1.543, p = 0.123). There was weak evidence of an association between sex and participation status (X^2 ^= 4.24 on 1 df, p = 0.039), with more male non-participants than expected (25 observed, 20 expected) and fewer female non-participants (31 observed, 36 expected).

**Table 2:** Characteristics of the 123 patients diagnosed as having IBS

	**Overall N 123, (100%)**	**Participants** **N 67, (55%)**	**Non-participants** **N 56, (45%)**	**Significance** **p = 0.039**
**Sex**				
**Male**	43 (35%)	18 (27%)	25 (44.5%)	

**Female**	80 (65%)	49 (73%)	31 (55%)	

**Median age (min-max)**	71 (20–97)	70 (28–92)	76 (20–97)	p = 0.342

**Age groups**				

**25–44**		7 (10.4%)	4 (7%)	

**45–64**		25 (37.3%)	14 (25%)	

**65–79**		25 (37.3%)	15 (26.7%)	

**> 80**		10 (14.9%)	22 (39.3%)	
**Education**				

**None**		6 (9%)	Not known in most	
**Primary**		50 (74.6%)	cases	
**Secondary**		11 (16.4%)		

### Old vs new diagnostic criteria

Of the 67 IBS patients that finally participated in the interview, 46 (69%, 95% CI: 58%–80%) fulfilled two or more of the Manning criteria by the time of interview. Thirty-two subjects (48%, 95% CI: 36%–60%) fulfilled the Rome II criteria, all of them also fulfilled the criteria of Manning. The modified Rome III questions/criteria were satisfied by 16 subjects (24%, 95% CI: 14%–34%), all of whom also fulfilled both Rome II and Manning criteria. Twenty-seven patients (40%, 95% CI: 29%–52%) satisfied the conditions for IBS according to the FGIDs questionnaire.

Poor agreement was found between the Rome III and the Manning criteria, kappa = 0.25 (95% CI: 0.12 to 0.38). Only moderate agreement was found between the Rome II and Rome III criteria, kappa 0.51(95% CI: 0.33 to 0.69). There was also poor agreement between the FGIDs questionnaire and the Manning, Rome II and the Rome III criteria with kappa = 0.30 (95% CI: 0.12 to 0.49), kappa = 0. 31 (95% CI: 0.08 to 0.53) and kappa = 0.24 (95% CI: 0.01 to 0.46) respectively. Gender and age were not statistically significant risk factors for the positive diagnosis of IBS with any of the diagnostic criteria.

### Co morbidity

Five (7.5%, 95% CI: 1.2%–13.8%) of the participants stated that they did not suffer from any gastrointestinal symptom in the last 12 months prior to the interview.

The investigation for co morbidity with other gastrointestinal disorders revealed 31 patients (46%, 95% CI: 34%–58%) experiencing GERD like symptoms according to the IDGP questionnaire. Within this group of patients 24 (77.4%) fulfilled the criteria of Manning, whereas 15 (48.4%) and 8 (25.8%) fulfilled the Rome II and III criteria respectively. Nine of the 67 patients (13%, 95% CI: 5% to 22%) patients had undergone cholecystectomy or experienced gall bladder problems in the past. Seven (10.4%, 95% CI: 2.5% to18%) patients had dyspepsia according to the IDGP questionnaire and one patient had FD according to Rome II. Four of the patients (6%, 95% CI: 0.3% to12%) had been diagnosed with cancer (1 gastric, 1 ovarian, 2 cervical).

Sixteen patients were suffering from one or more gastrointestinal symptom (24%, 95% CI: 14% to 34%) without fulfilling any of the IBS criteria. Symptoms more frequently than 6 times per year were reported by 59 (88%) of the participants whereas 3 (0.4%) had symptoms less frequently.

### The main findings of the study

In our study population more patients fulfilled Manning's criteria, fewer the Rome II and even fewer the Rome III criteria which proved the most restrictive. In previous studies the criteria of Manning and the Rome III criteria were found more sensitive in diagnosing IBS patients in primary care compared to Rome II. [15-17] The complexity of questions about the duration of symptoms might have played an important role for the difference between the Rome II and III criteria. It is also supported that criteria that are based on the frequency of symptoms have lower prevalence values compared to criteria based on the presence of symptoms. [18,19] Our findings indicate that IBS diagnosis in rural areas of Crete has not been based on complex criteria. In the same vein, the FGIDs questionnaire revealed fewer patients as having IBS than the Manning and Rome II criteria and showed low agreement compared with all the criteria. This questionnaire was expected to be more restrictive in the primary care population as there is a strong argument that primary care patients have different disease characteristics than outpatients. [19,20]

High co-morbidity with GERD like symptoms was noted. The observed rate in our study (46%) was among the highest reported according to a review of the international literature. [21] It is difficult to explain this prominent overlap and although both conditions are highly prevalent, the overlapping symptoms are lately attributed to a possible common disease process. [22] Co morbidity with dyspepsia was relatively low (10.4%) compared with other studies. [20]

### The study findings in the light of other studies

Criteria developed by specialists have been criticized for low performance in primary care.[6,23,24] Skepticism as to the degree of relevance of Rome diagnostic criteria for IBS with everyday clinical primary practice is developing and authors have suggested that the next consensus meeting on IBS should be interdisciplinary. [15,25] Our results are in agreement with international literature on the low application of diagnostic criteria for IBS and especially the Rome II.[5] The Rome III criteria are considered as less restrictive and thus closer to primary care reality,[16,17] but in our study this role was not verified. In the Greek primary setting the number of visits to the doctor due to IBS was found low [8] compared to international data. In another study from Crete, again, IBS patients reported that they did not visit the PHC centre for their IBS problems frequently. [26] All data form a puzzle showing that in IBS patients in rural areas of Crete, both actual and as perceived by individuals, symptoms are rather underestimated. Further research is needed to confirm it.

### Limitations of the study

Our study used the database of IBS patients identified in medical records in a retrospective research. Information as to what criteria were applied by primary care doctors was not available. In most cases the diagnosis alone was the only available data. Also poor demographic data entries resulted in high numbers of excluded or non-participating patients limiting in this way the strength of the results. For the majority of the non participating patients there were no available data about the presence of gastrointestinal symptoms. Thus a potential selection bias could be addressed. It should also be noted that although Cohen's kappa statistic is an extremely widely used measure of agreement at the present time in the biomedical literature, certain "paradoxes" in its interpretation have been noted in relation to unbalanced marginal totals, and also its dependence on the prevalence of the condition [27].

Another limitation was the use of modified questions matching the Rome III instead of the actual Rome III criteria for a retrospective comparison. A similar approach was attempted in another study the results of which followed the pre existing research on Rome III. [14] Our study provides a hint on the application of the Rome III in IBS patients in rural Crete at a time where no other information is available.

The 6.4 years interval between the first diagnosis and the structured interview is another limitation as it could allow changes and overlaps with other gastrointestinal diseases, a finding common in IBS patients. [28]This interval did not allow a direct comparison between the criteria and doctor's diagnosis, but the retrospective comparison between criteria at the time of interview was possible.

### Implementation to practice and suggestions for future research

The low agreement between older and new criteria and the tendency for greater fulfillment of the criteria of Manning; reveal the necessity for a different approach to the diagnosis of IBS in primary care in rural areas of Greece. This approach has been also highlighted in a consensus development for the diagnosis of IBS in primary care. [18] Clinical manifestations of IBS and co morbidity with other gastrointestinal diseases; both in primary care patient and the general population in rural Greece; should also be investigated in order to obtain a clear picture of the syndrome.

## Conclusion

In Greek primary care, international diagnostic criteria display low agreement for the diagnosis of IBS. Amongst these, the newest criteria display worse results than expected. Questionnaires and criteria deriving from tertiary hospitals or expert's consensus meetings seem to be applied with difficulty in rural primary care where symptoms are underestimated by patients and complicated questions can be confusing.

## Abbreviations

IBS: Irritable Bowel Syndrome; IDGP: Identification of Dyspepsia in the General Population questionnaire; FGIDs: Identification of Functional Gastrointestinal Diseases questionnaire; GERD: Gastro Esophageal Reflux Disease; PHC: Primary Health Centre

## Competing interests

The research programme received a grand from AstraZeneca Sweden.

## Authors' contributions

CL, HC, IM, and FA conceived the idea. CL supervised the collection of data. FA collected and analysed the data, performed the interviews. JM performed all statistical analysis. FA, JM and CL prepared the first draft. All authors read and approved the final version of the manuscript.
